# Hotspots and Frontiers of Host Immune Response in Idiopathic Pulmonary Fibrosis: A Bibliometric and Scientific Visual Research from 2000 to 2022

**DOI:** 10.1155/2023/4835710

**Published:** 2023-04-19

**Authors:** Shirong Li, Pengyue Zhao, Chao Wang, Yun Xia, Haoyan Wang, Wenjie Qi

**Affiliations:** ^1^Department of Infectious Disease, Beijing Friendship Hospital, Capital Medical University, Beijing 100050, China; ^2^Department of General Surgery, First Medical Center of Chinese PLA General Hospital, Beijing 100853, China; ^3^Department of Anesthesiology, Zhongnan Hospital of Wuhan University, Wuhan 430000, China; ^4^Department of Respiratory, Beijing Friendship Hospital, Capital Medical University, Beijing 100050, China

## Abstract

**Background:**

Idiopathic pulmonary fibrosis (IPF) is a disease with significant morbidity, progressive deterioration of lung function till death, and lack of effective treatment options. This study aims to explore the global research trends in IPF and immune response to predict the research hotspot in the future. *Materials and methods*. All related publications on IPF and immune response since the establishment of diagnostic criteria for IPF were retrieved using the Web of Science (WOS) database. VOSviewer, GraphPad Prism 6, CiteSpace version 5.6. R5 64-bit, and a bibliometrics online platform were used to extract and analyze the trends in relevant fields.

**Results:**

From March 1, 2000, to September 30, 2022, a total of 658 articles with 25,126 citations met the inclusion criteria. The United States ranked first in number of publications (*n* = 217), number of citations (*n* = 14,745), and H-index (62). China ranked second in publications (*n* = 124) and seventh and fifth for citation frequency and H-index, respectively. The *American Journal of Respiratory and Critical Care Medicine* (impact factor = 30.528) published the most articles in the field. The author Kaminski N. from the United States was the most influential author with 26 publications and an H-index of 24. Among the 52 keywords that co-occurred at least 20 times, the main keywords were concentrated in “Inflammation related” and “Biomarker related” clusters. “biomarker” (AAY 2018.64, 25 times) was a newly emerged keyword.

**Conclusions:**

The United States has an unequivocal advantage in IPF and immunization, but China shows a faster developing trend. The *American Journal of Respiratory and Critical Care Medicine* should be prioritized for leading articles. This study indicates that exploration of ideal immune-related biomarkers to provide evidence for the clinical work of IPF might be a hotspot in the near future.

## 1. Introduction

Idiopathic pulmonary fibrosis (IPF) is a chronic, fatal pulmonary disease of unknown etiology characterized by inexorable respiratory function failure, eventually leading to death [1, 2]. More than 3 million people suffered from IPF worldwide before 2015 [3], and its incidence is increasing [4] due to harmonized nomenclature [5], standardized diagnostic criteria [6], and improved diagnostic tools [7]. With an aging population, the impact of IPF on the medical system and its burden on the social economy will worsen [8, 9]. Currently, Pirfenidone and Nintedanib are the only two options approved by the FDA, and evidence-based guidelines recommend them as medicines for treatment of IPF alone or in combination [8, 10, 11]. However, these two medications cannot reverse the decline of lung function, disease progression, and death [12]. The prognosis of IPF is extremely poor with an average survival time of only 2.5–3.5 years from diagnosis [13]. When medical options have been exhausted, lung transplantation became the only treatment available for IPF. But in fact, lung transplantation benefits only a small number of IPF patients due to limitations including patient age, comorbidities, and scarcity of donor organs [12, 14]. Challenges in IFP diagnosis and treatment are due to a lack of deep understanding of the pathogenesis [8].

Initially, IPF was considered to be associated with inflammation because of imaging and pathological findings. As such, corticosteroids and immunosuppressive/cytotoxic agents alone or in combination have been recommended treatment options [15]. However, subsequent studies found that immunosuppressive therapy did not provide adequate benefit and even increased the risk of death [16, 17]. Thus, the hypothesis that inflammation leads to fibrosis has been questioned. Recent studies found that immune dysregulation may play a profibrotic role via immune regulation, rather than directly causing IPF [18, 19]. Intrinsically, immune dysfunction has been related to various immune cells, such as peripheral blood mononuclear cells (PBMCs) [20], dendritic cells (DCs) [21], and pulmonary macrophages [22]. In addition, clinical guidelines still recommend corticosteroids for patients with acute exacerbations of IPF, albeit weakly [10]. Thus, a better understanding of the progress of research on immunity in IPF, an in-depth study of immune regulation mechanisms, and a search for new therapeutic targets to reverse immune disorders have attracted worldwide attention. An overall analysis of the published literature would help to quickly identify the research hotspots and development trends in host immune response associated with IPF.

Bibliometrics is a mathematical and statistical research method that analyzes the distribution structure and changing rules of a certain field quantitatively based on the published information. Bibliometrics has been reported to be used for subject trend analysis, frontier identification, and evolution prediction [23, 24]. Bibliometrics is also used to evaluate the research contributions and collaborative relationships in a specific area across countries, institutions, journals, scholars, and articles [25], which can guide scholars to choose appropriate journals to publish their research results by analyzing citations and publication patterns [26–28]. More importantly, bibliometrics can also be used as a scientific basis for developing and implementing disease treatment guidelines [29].

Relying on the Web of Science (WOS) database, bibliometric analysis was adopted for the first time to quantitatively analyze the IPF and host immune response literature and evaluate the contributions and cooperation of different countries, institutions, and researchers in the field of IPF. The aim of this study was to reveal the current status and research trends in IPF and immune response, and predict future research hotspots to provide a theoretical basis for the pathogenesis and clinical research of IPF.

## 2. Materials and Methods

### 2.1. Data Source and Retrieval Strategies

**Figure 1:**
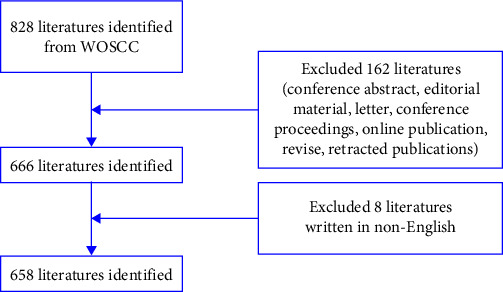
Detail flowchart of retrieval, screening, and enrollment process using the WOS in this study.

Science Citation Index-Expanded (SCI-E) of Thomson Reuters' WOS is considered to be the best database for bibliometric analysis and it is widely used in scientific research [30, 31]. Thus, WOS Core Collection (WOSCC) was applied for the online literature retrieval in this study. The definition of IPF was constantly revised and advanced [32]. The initial international consensus statement defining the normalized diagnosis of IPF was produced co-operatively by the American Thoracic Society, the European Respiratory Society, and the American College of Chest Physicians, and it was published in February 2000 [15]. Thus, the timespan selected to retrieve the publications was from March 1, 2000, to September 30, 2022. To avoid alteration of the data involved during the database updates, data collection and download were completed within a single day on September 30, 2022. The retrieval strategy was as follows: TI = (Idiopathic Pulmonary Fibrosis) OR (Pulmonary Fibrosis, Idiopathic) OR (IPF) OR (cryptogenic fibrosing alveolitis) OR (CFA) OR (lone CFA) OR (idiopathic interstitial pneumonia) OR (Fibrosing Alveolitis, Cryptogenic) OR (Cryptogenic Fibrosing Alveolitides) OR (Fibrosing Alveolitides, Cryptogenic) OR (Pulmonary Fibrosis, Idiopathic) AND TS = immunology OR (immune response) OR (immunologic response) OR (immune dysfunction) OR immunosuppression OR macrophage OR monocyte OR neutrophil OR (natural killer cell) OR (NK cell) OR (innate lymphoid cell) OR (innate lymphocyte) OR ILC OR (dendritic cell) OR DC OR (T lymphocyte) OR (T cell) OR (regulatory T cell) OR (Treg) OR (B lymphocyte) OR (B cell) AND LANGUAGE: (English) AND DOCUMENT TYPES (Article OR Review). Data download and retrieval were independently conducted by two authors. Ethics committee approval was not required because all data were obtained from public databases. Details of literature inclusion, screening, and exclusion are shown in Figure 1.

### 2.2. Data Collection

All data extracted and downloaded from qualified publications included titles, keywords, countries/regions, institutions, authors, publication dates, published journals, citation frequency, and H-index, and this process was independently performed by two authors (LSR and ZPY). Microsoft Excel 2016 (Redmond, Washington, USA), GraphPad Prism 8 (GraphPad Prism Software Inc., San Diego, CA, USA), VOSviewer software (Leiden University, Leiden, Netherlands), CiteSpace version 6.1. R3 and bibliometrics online platform (http://bibliometric.com/) were applied to qualitatively and quantitatively analyze and construct a visual network of relationships among five entities: countries, institutions, journals, researchers, and keywords.

### 2.3. Bibliometric Analysis

In this study, three evaluation parameters were used in the bibliometric analysis. The latest impact factor (IF), identified as an important index to measure academic impact of a journal or research output, was acquired from the latest issue of Journal Citation Reports (JCR) published in 2022 [24, 25]. The H-index, defined as the H number of papers published by a country/scholar with at least H number of citations and known as a well-established quantitative indicator to evaluate the academic output of a country/research [33], was obtained from the WOS database. Relative research interest (RRI) was used to indicate the ratio between the published number in a specific field and publications' overall fields per year. VOSviewer is a software tool that was used to construct and visualize bibliometric networks [34], and it is used for keyword network construction and cluster analysis. Average appearing year (AAY) was applied to quantify the relative novelty of the keyword by coloring based on the chronological order of appearance.

## 3. Results

### 3.1. Global Growth Trends of Publications

**Figure 2:**
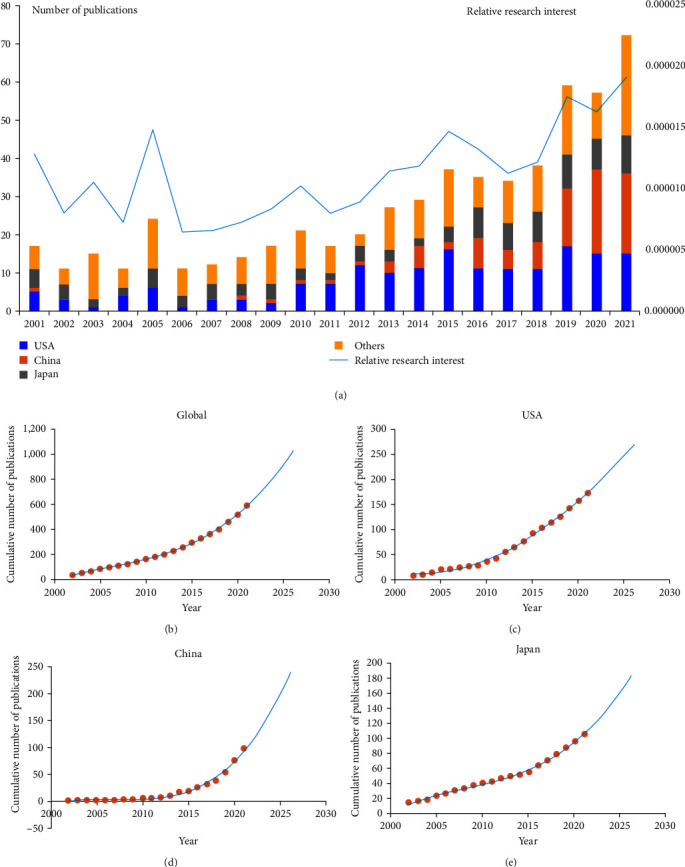
Global growth trends of publications on IPF and immune response. (a) Bar chart showing the number of publications per year for the world and the top three countries; the timeline of relative research interest in IPF and immune response ( ^*∗*^means “×”). (b) A model fitting curve of growth trends across the globe, United States (c), China (d), and Japan (e).

As presented in Figure 1, a total of 658 publications from March 1, 2000, to September 30, 2022, that met the inclusion criteria for this study were used for subsequent investigation. The number of publications worldwide showed an upward trend (Figures 2(a) and 2(b)). When publications in all fields were taken into account, the global interest in a certain field was measured by the RRI value, which fluctuated around 0.001% from 2001 to 2018, nearly doubled in 2019, and showed a continuous upward trend (Figure 2(a)). A total of 188 articles were published in the 3 year period from 2019 to 2021, accounting for 28.6% of the total published articles in the last 22 years. The United States, China, and Japan were the top three countries in terms of publications on IPF and immune response. Up to 2021, the graph of annual publications showed that the overall publication trends of the top three countries were consistent with the global trends basically (Figures 2(b) and 2(e)). In the past 3 years, China showed the highest growth trend (Figure 2(d)).

### 3.2. Contributions of Countries/Regions to Global Research on IPF and Immune Response

**Figure 3:**
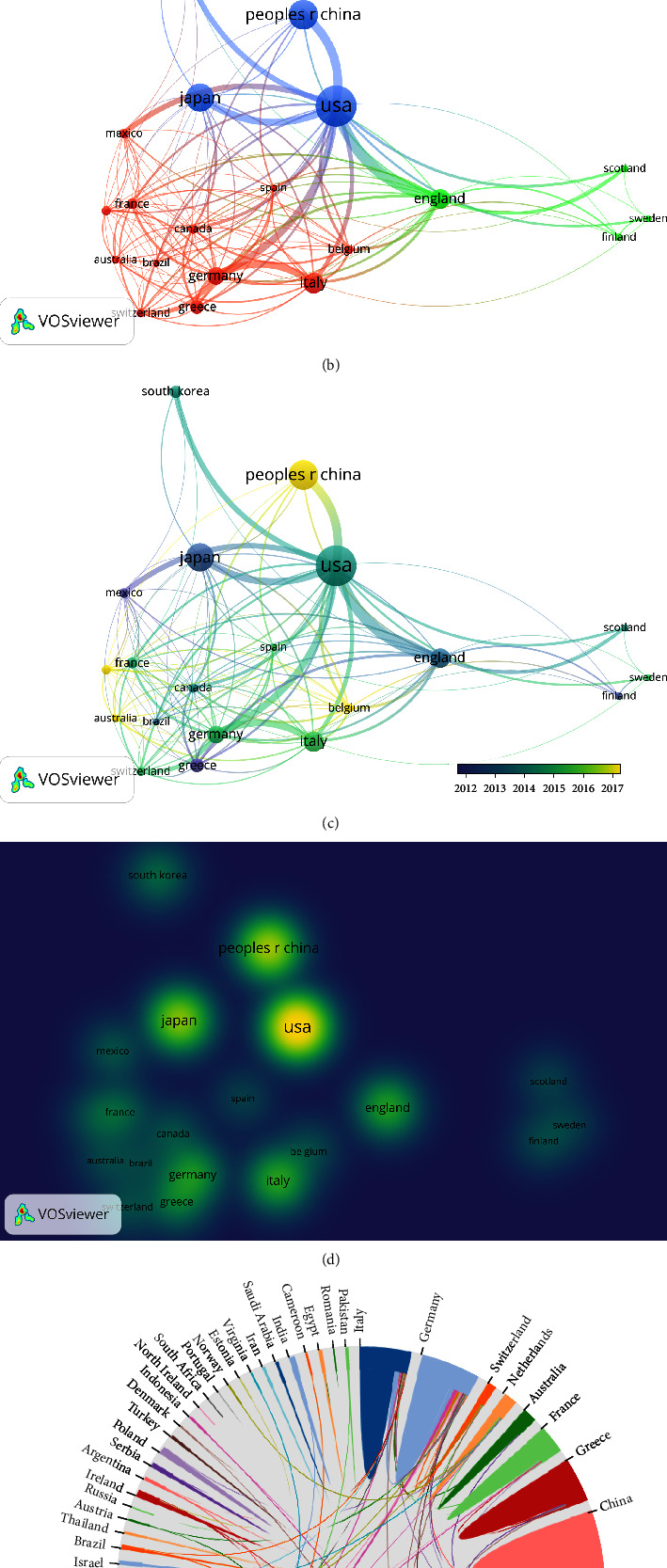
Contributions of different countries/regions in the field of IPF and immune response. (a) The number of publications, total number of citations (×0.01), and H-index (×2) in the top 15 countries/regions. (b) Map of the international cooperation network of the top 20 countries/regions. The circle size represents the number of publications. (c) Countries/regions were colored based on the average publication time. Blue color represents early years, and yellow color represents more recently. (d) The thermodynamic chart of countries/regions weight in this field established using VOSviewer. (e) A pie chart of the contributions and cooperation of different countries/regions established using the bibliometrics online platform.

The WOS citation results showed that 658 articles related to IPF and immune response were cited 25,126 times (excluding self-citation of 2,212 times), with an average citation frequency of 38.19 times per article and an H-index of 75. Among the analyzed data, 217 (32.98%) publications from the United States accounted for the highest number of publications. They got 14,745 citations, accounting for 58.68% of the total citations, with an average citation frequency of 67.95 times per paper and an H-index of 62. China ranked second with 124 articles published in this field. However, the number of citations and H-index were 1,595 (6.35%) and 23, ranking seventh and fifth, respectively. Japan and Italy ranked third and fourth in terms of the number of publications with 110 (16.72%) and 62 (9.42%) articles, respectively. However, the number of citations was 3,623 (14.42%) and 1,742 (6.93%), respectively, which were higher than those of China. The H-index values of Japan, England, and Germany were 35, 29, and 24 respectively, which were also higher than those of China, although their publication volume was lower than that of China (Figure 3(a)).

Visualized the top 20 countries with the most relevant publications, the United States was at the core of the thermodynamic map and cooperative relationship network with the largest cycle, reflecting the fact that the United States published the highest number of articles and had the closest cooperation with other countries, especially with Japan and China in Asia, as well as Britain and Germany in Europe (Figure 3(b)–3(d)). These countries/regions were colored based on the average publication time. Blue color represents early years, and yellow color represents more recently. China (AAY 2018.75) was the latest influential country in this area and is colored in bright yellow (Figure 3(c) and *Supplementary 1*). The online bibliometrics platform that built the contribution of all countries and the mutual cooperation relationship visualizes the advantages of the United States and China concerning the number of publications and the abundance and efficiency of international cooperation relationships of the United States (Figure 3(e)).

### 3.3. Contributions of Institutions and Journals to Global Research on IPF and Immune Response

**Figure 4:**
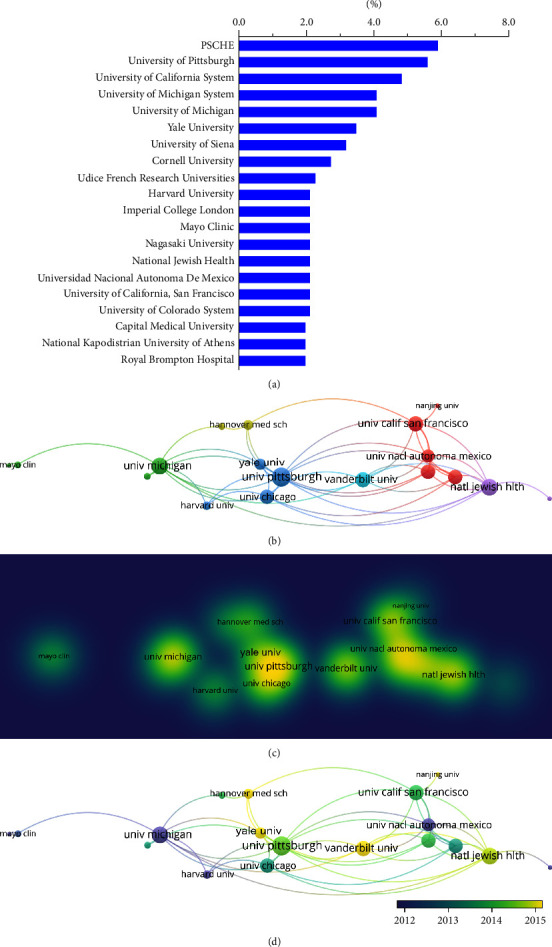
Contributions of different institutions in the field of IPF and immune response. (a) The top 20 institutions ranked by the number of publications. The *X*-axis represents the percentage of the number of publications by the institution to the total. (b) Map of the international cooperation network of the top 20 institutions with at least eight publications in IPF and immune response. The circle size, the complexity of the network structure, and the thickness of the lines represents the importance in this field. (c) Density visualization of institutional weight in this field. (d) Institutions were colored based on the average publication time. Blue color represents published in the early years, and yellow color represents published more recently.

The retrieved results of WOS showed that in terms of the number of papers published among the top 20 institutions, 12 institutions were from the United States, one was from Japan, and one was from China. The Pennsylvania Commonwealth System of Higher Education (PCSHE), University of Pittsburgh, and University of California System comprised the top three institutions with 39 (5.93%), 37 (5.62%), and 32 (4.86%) publications, respectively (Figure 4(a)).

The cooperative relationships of the main organizations were quantified and visualized using Vosviewer. The minimum number of documents of an institution was set at 8, and we observed the distribution of the institutions engaged in IPF and immune response. The University of Pittsburgh in the United States was located at the center of the map with the largest node and the most complex and bulky network structure because of its greatest influence in this field (Figures 4(b) and 4(c)). Furthermore, the institutions were colored by VOSviewer according to the average time taken to make an impact; the blue color indicates the early years, and the yellow color represents relatively recent. Among them Hannover University Medical School (AAY 2020.75) in Germany, Nanjing University (2018.75) in China, and Yale University (AAY 2015.33) in the United States were the emerging institutions that contributed publications and cooperation in the field of IPF and immune response (Figure 4(d) and *Supplementary 2*).

**Figure 5:**
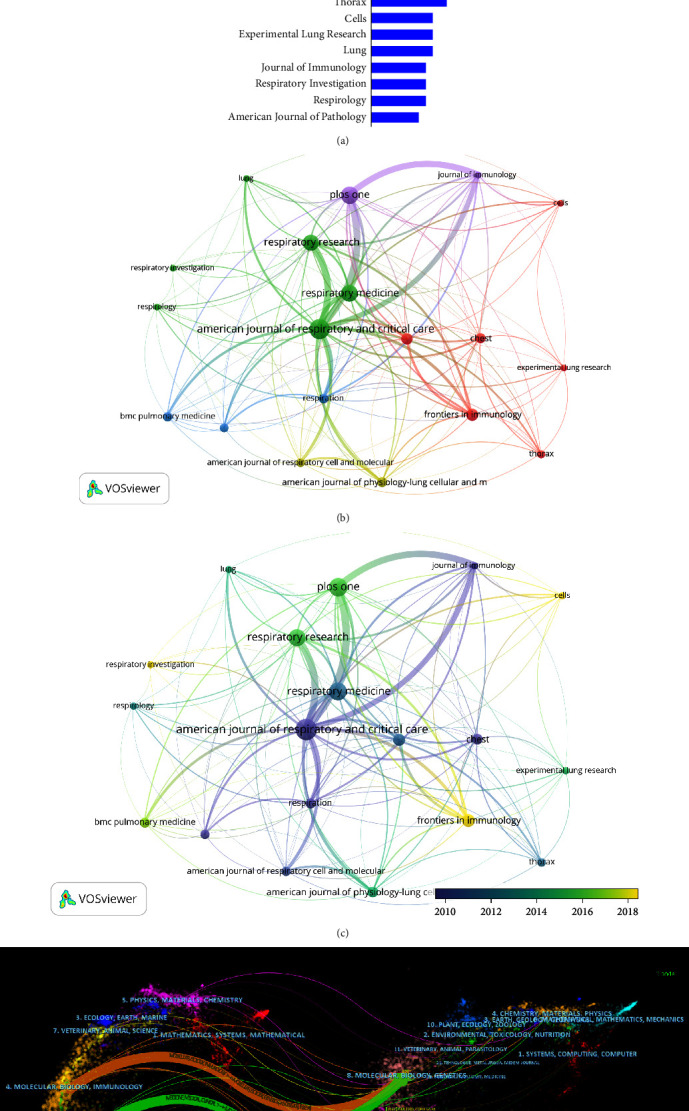
Contributions of different journals in the field of IPF and immune response. (a) The top 20 journals ranked by the number of publications. The *X*-axis represents the percentage of the number of publications by the institution to the total. (b) Map of the citation network of the top 20 journals with at least eight publications in IPF and immune response. The circle size represents the number of publications included. The lines between circles represent the cooperation weight. (c) Journals were colored based on the average citation time. Blue color represents in the early years, and yellow color represents more recently. (d) The dual-map overlay of journals related to IPF and immune response. The citing journals are on the left, and the cited journals are on the right. The color line between them indicates the cited relationship.

With respect to the most influential journals in the field of IPF and immune response, 179 papers (27.20%) related to IPF and immune response were published in the top 10 journals (Figure 5(a)). The *American Journal of Respiratory and Critical Care Medicine* (IF = 30.528) published 32 articles in this field and ranked first in terms of the number of publications, with 3,723 citations, accounting for 14.82% of the total citations. *PLOS ONE* (IF = 3.752) and *Respiratory Medicine* (IF = 4.582) ranked second and third, respectively, with 27 and 26 publications. The citation analysis using VOSviewer showed that the *American Journal of Respiratory and Critical Care Medicine* (IF = 30.528) was at the center of the map with the biggest cycle and the most complex network line structure due to the highest number of publications included and cooperation weight (Figure 5(b)). While *Cells* (AAY 2021.44) and *Frontiers in Immunology* (AAY 2020.18), colored bright yellow, were the two latest journals that focused on this field and made an impact (Figure 5(c) and *Supplementary 3*). As shown in Figure 5(d), in the field of IPF and immune response, research related to molecular/biology/immunology mainly cited journals in the field of molecular/biology/genetics, while research related to medicine/medical/clinical mainly cited journals related to molecular/biology/genetics and health/nursing/medications.

### 3.4. Authors Who Published Research on IPF and Immune Response

Kaminski N. from Yale University was the most published author in the IPF and immune response field, publishing 26 papers and receiving 2,446 citations. Bargagli E. from Siena University Hospital and Selman M. from Instituto Nacional de Enfermedades Respiratorias Ismael Cosío Villegas ranked second and third with 17 and 16 publications, respectively. The top 10 most productive authors published a total of 152 articles, accounting for 23.10% of all publications. Among them, six authors were from the United States, two were from Italy, and two were from Mexico. Notably, Selman M. and Pardo A. from different institutions in Mexico ranked first (2,974 citations) and second (2,898 citations), respectively, among all authors in terms of citations (Table 1). The top 10 highly cited papers related to IPF and immune response and their corresponding authors are shown in Table 2.

### 3.5. Analysis of Keywords and Hotspots in Publications on IPF and Immune Response

**Figure 6:**
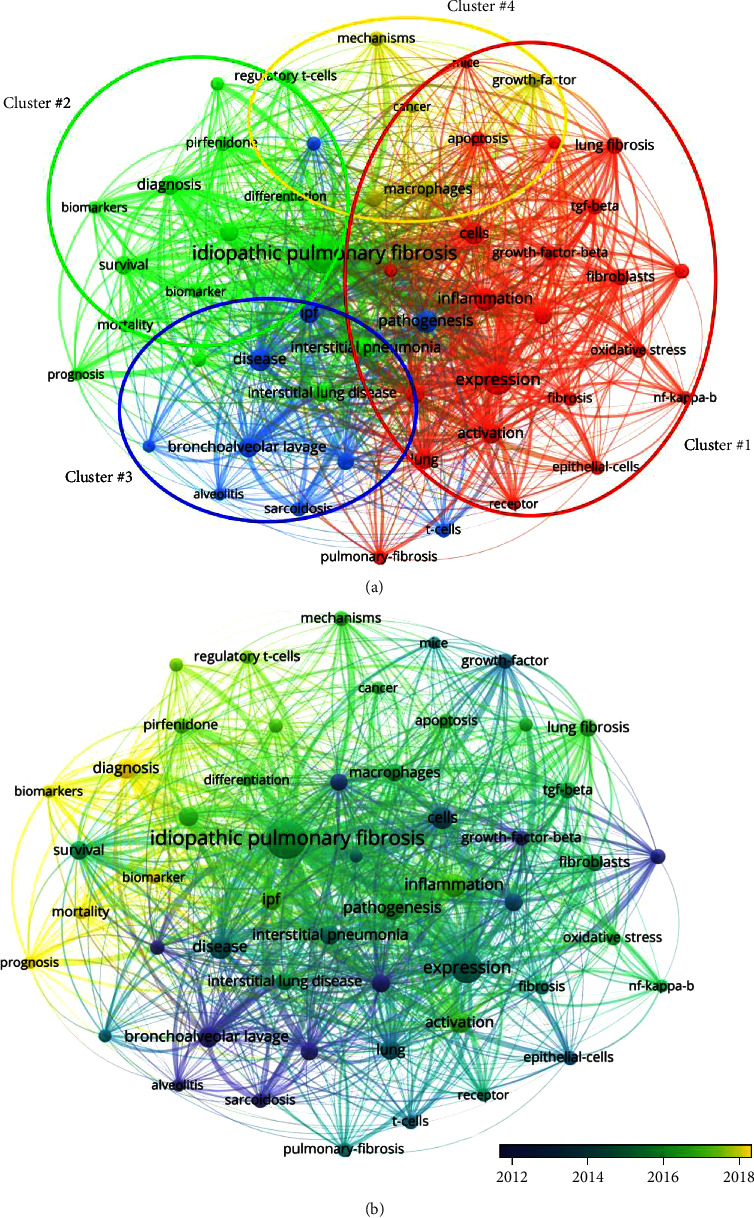
A cluster analysis diagram of keywords in publications of IPF and immune response. (a) Mapping of the keywords that appeared at least 20 times. Four clusters were automatically generated using VOSviewer software and defaulted to different colors. Each cluster was artificially named “inflammation related” (right in red), “biomarker related” (left in green), “disease related” (down in blue), and “innate immune mechanism” (up in yellow). The circle size represents the frequency of occurrence. (b) Keywords were colored based on the average time of occurrence. Blue color represents in the early years, and yellow color represents more recently.

To explore the main focus of this field, based on the 658 publications on IPF and immune response, we extracted 2,934 keywords from the titles and abstracts of all publications and performed an analysis of 52 keywords that co-occurred at least 20 times using VOSviewer software (Figure 6(a)). The diameter of each keyword circle indicates its co-occurrence frequency. The 52 keywords were grouped into four clusters in an acquiescent manner, and they were artificially named as follows: “Inflammation related” (cluster #1, red in the map), “Biomarker related” (cluster #2, green in the map), “Disease related” (cluster #3, blue in the map), and “Innate immune mechanism” (cluster #4, yellow in the map). In each cluster, there were keywords with the highest co-occurrence frequency. For example, the most frequently occurring keywords in the “Inflammation related” cluster were “expression” (142 times), “inflammation” (76 times), “cells” (71 times), and “activation” (57 times). The most common keywords in the “Biomarker related” cluster were “idiopathic pulmonary fibrosis” (252 times), “diagnosis” (57 times), “acute exacerbation” (52 times), and “survival” (48 times). The main keywords in the “Disease related” cluster were “disease” (77 times), “pathogenesis” (75 times), “ipf” (58 times), “bronchoalveolar lavage” (56 times), and “bronchoalveolar lavage fluid” (40 times). The keywords “macrophages” (46 times), “pulmonary fibrosis” (41 times), and “growth factor” (32 times) appeared frequently in the “Innate immune mechanism” cluster. The details of these 52 keywords are shown in *Supplementary 4*.

To present the time of appearance of each keyword, VOS software colored each keyword according to the average time of co-occurrence, with bright yellow representing the latest occurrence and blue representing the earliest occurrence (Figure 6(b)). Analysis of these 52 keywords showed that “sarcoidosis” (AAY 2010.23, 31 times), “alveolar macrophages” (AAY 2011.53, 47 times), “growth-factor-beta” (AAY 2011.6, 30 times), and “bronchoalveolar lavage fluid” (AAY 2011.68, 40 times) appeared relatively early and were located in clusters #1 and #3. However, “biomarker” (AAY 2018.64, 25 times), “mortality” (AAY 2018.48, 26 times), “diagnosis” (AAY 2018.30, 57 times), “prognosis” (AAY 2018.05, 22 times), and “biomarkers” (AAY 2017.74, 23 times) were relatively new and frequently occurring keywords. Interestingly, all of these newly co-occurred keywords were included in cluster #2, indicating that cluster #2 could attract more attention from researchers. On the other hand, CiteSpace was used for cluster analysis of keywords and classification according to the characteristics of keywords. The top 10 largest clusters are shown in *Supplementary 5*. Furthermore, the chronological order of keywords and co-occurrence were visualized using CiteSpace (*Supplementary 5*).

## 4. Discussion

### 4.1. Research Trends in IPF and Immune Response

This study clarified that the trend in global number of publications related to IPF and immune response has increased since the diagnostic criteria for IPF were published by the American Thoracic Society in 2000 [15]. The United States ranked first in the world in terms of the number of publications, the number of citations, and the H-index. This fully demonstrates the outstanding contribution of the United States in the field of IPF and immune response. Consultation with the literature on IPF revealed that several revisions of the diagnostic criteria and treatment principles of IPF were jointly published by scholars from the United States and Europe [15, 32, 35], indicating that the United States and Europe have given more attention to this field compared with other countries. Moreover, the United States has a considerable advantage in immune research, which is supported by the number of articles and is likely related to comprehensive national strength, professional researchers, sufficient research funds, and extensive international cooperation [23, 29, 30]. Thus, the United States is at the leading level in this field due to several advantages.

With respect to citations and H-index, Japan and England followed the United States closely (Figure 3(a)). China ranked second in the number of publications in this field; however, it lagged behind Japan and the United Kingdom in terms of citations and H-index, ranking seventh and fifth, respectively. The discrepancy between the number of publications in China, citations, and H-index has also been revealed in other studies [23, 29, 30], which could be attributed to several factors. The first reason is that China entered relatively late into the IPF and immune-related fields. Before 2013, only sporadic articles were published, and then the number gradually increased (Figure 2(a)). From 2019, the number of articles published in this field started to increase significantly, and China caught up with other countries and even exceeded their number of articles. The number of citations lags behind the number of publications. Therefore, the growth of citations in China will take more time. Second, regardless of the influence of gender and age, the incidence of IPF in China is lower than that in the United States and most other countries in the world [4]. Further, sporadic cases were scattered in different places, and diagnostic challenges increased the difficulty to carry out standardized clinical research. Third, China may need to expand international exchanges and cooperation in this field based on high-level scientific research to promote its own development and common progress (Figures 3(b) and 3(e)).

In terms of institutions, one paper may be completed by authors from several universities or medical institutions, and each university or medical institution may belong to different systems. The analysis of institutions by different software is not the same, resulting in inconsistent results. According to WOS results, the PCSHE was the most published institution in the field of pulmonary fibrosis and immune response with the University of Pittsburgh ranking second (Figure 4(a)). PSCHE is a “state-related” institution composed of the universities within the state, including the University of Pittsburgh (https://www.passhe.edu/Pages/default.aspx). Therefore, for an independent institution, the University of Pittsburgh was in a dominant position in this field. This conclusion was confirmed by Vosviewer (Figures 4(b) and 4(c)). From another perspective, most of influential institutions in this field were from the United States and Europe (Figure 4(a)–4(c)). Considerable advances in this field and extensive international cooperation between the United States and some European countries have also promoted the development of several guidelines or guidances, which provide reliable evidence for clinical and scientific research [5, 10, 15, 32, 36]. In addition, Hannover Medical School in Germany (AAY 2020.75) and Nanjing University in China (AAY 2018.75) are the latest institutions to focus on this field and have had an impact on IPF and immune response research (Figure 4(d)). In a certain sense, adequate international cooperation can effectively improve the influence of countries, institutions, scholars, and publications. Our analysis indicates that China needs to increase international exchange and cooperation while improving the quality of research in the future.

The analysis of journal contributions and citations can assist researchers to understand the importance of a field, and it can provide important assistance for efficient retrieval of academic frontier literature, writing, or publishing research results. According to this study, current outstanding achievements in IPF and immune response research were mainly published in journals related to the respiratory system, cell biology, and immunology. Among the top 10 journals in terms of publication volume, three journals had an impact factor of more than 10, and three journals had an impact factor between 5 and 10 (Figure 5(a)). The *American Journal of Respiratory and Critical Care Medicine* published the highest number of articles with the most citations. It is worth noting that high-quality papers on IPF and immune response have been published in journals with high impacts, such as the *Lancet Respiratory Medicine* (IF = 102.642) and the *New England Journal of Medicine* (IF = 176.079). Thus, the medical community and academics are acknowledging the importance of IPF and immune response. *Cells* and *Frontiers in Immunology* were the latest journals to have an impact in this field, providing more publishing opportunities for the research in IPF and immune response field.

Regarding the most productive and influential author, Kaminski N. from Yale University School of Medicine in the United States ranked first in the field of IPF and immune in terms of publications and H-index. Kaminski N, who published 15 articles with impact factors greater than 30 and participated in the research entitled “Small Airways pathology in Idiopathic Pulmonary Fibrosis: A Retrospective Cohort Study” published in the *Lancet Respiratory Medicine* (IF = 102.642), was quantified as one of the most influential authors in IPF and immune response. Selman M. and Pardo A. from Mexico ranked first and second in terms of citation frequency because they had published many high-level articles in influential journals (Table 1).

### 4.2. Research Focus on IPF and Immune Response

The number of times an article is cited and the impact factor value of the published journal objectively reflect the academic influence of the research in a field. The top ten cited articles in the fields of immune response and IPF were published in journals with an impact factor >5 and half of these had an impact factor >30 (Table 2). The research content mainly focused on the mechanism and treatment of IPF, which also included the difficulty in overcoming IPF, an incurable disease. Among these articles, Selman M and Pardo A from Mexico participated in the research of four publications, and Kaminski N. from the United States participated in two studies along with the first two authors. These three authors were also the top three cited authors. This fully illustrates the importance of collaboration between authors. “Idiopathic pulmonary fibrosis: Prevailing and evolving hypotheses about its pathogenesis and implications for therapy”, being the most cited paper, was published in *Annals of Internal Medicine* (IF = 51.598). This article summarized the relevant literature from 1965 to 2000. Based on the evidence that inflammation was not a significant pathological manifestation of IPF, inflammation was determined as unnecessary for fibrosis and was not associated with the clinical stage and prognosis, and anti-inflammatory treatment could not improve the outcomes. Thus, the article concluded that inflammation was not a requisite for IPF [37]. The second paper in the list was a review on IPF published in the *New England Journal of Medicine* (IF = 176.079) in 2001, which proposed a role for the type of inflammatory response in regulating tissue damage, fibrosis, or both during the evolution of IPF. It was pointed out that an increase in the Th2 cytokine interleukin- (IL-) 13 in IPF could be used as a marker of the transition to a Th1 inflammatory response mediated by immunomodulators, such as interferon *γ* [38]. This theory was supported by the fourth paper on the list. This article cited a theory for the role of IL-13, as well as IL-1*β*, tumor necrosis factor-*α*, and other profibrotic mediators. It also proposed that a cascade amplification reaction played an important role in the pathogenesis of IPF, among which IL-13 could promote the proliferation of fibroblasts and synthesis of extracellular matrix (ECM) [39]. The sixth paper addressed the antifibrotic mechanism of osteopontin [40], which was thought to primarily exert an antifibrotic Th1 effect on T lymphocytes [41]. In addition, three papers clarified that IPF was caused, at least in part, by the imbalance between matrix metalloproteinase (MMP) family members, and that MMP expressed by immune cells played an important role. For example, MMP-1 was also observed in alveolar macrophages, MMP-9 in neutrophils, tissue inhibitors of metalloproteinase-1 (TIMP-1) and TIMP-3 in interstitial macrophages, and TIMP-4 in plasma cells [40, 42, 43]. These articles supported the notion that immune cells and inflammatory cytokines take a crucial role in the pathogenesis of IPF and might act as potential therapeutic targets for IPF in the future.

There is no doubt that the keywords with the highest co-occurrence frequency are considered the focus and difficulty of this field and are being widely studied. The analysis of keywords by VOSviewer showed that in addition to the name and location of IPF, such as “idiopathic pulmonary”, keywords involved in the pathogenesis, such as “expression”, “inflammation”, “pathogenesis”, “cells”, and “activation”, occurred most frequently. All these findings confirm that exploring the role of immunity in the pathogenesis of IPF remains the focus of current research.

With respect to the latest hotspot, the latest keywords with high co-occurrence are generally regarded as the research hotspots in this field. The term “biomarker” was the most newly occurring keyword. In addition, “mortality”, “diagnosis”, “prognosis”, “biomarkers”, and “regulatory t-cells” were the other five most emerging keywords among the top ten keywords, and these were concentrated in the “Biomarker related” cluster. The term “inflammation” in the “Inflammation related” cluster remains a major concern in the field (Figures 6(a) and 6(b)). These data indicate that being an incurable disease, rapid and clear diagnosis, effective treatment, and improved prognosis are currently the focus of research on IPF. Although an in-depth study of the pathogenesis is the theoretical basis for clinical work, there is an urgent need to identify effective biomarkers, especially immune-related biomarkers, closely related to the mechanism, diagnosis, and assessment of treatment response in the field. Therefore, it is not difficult to speculate that the exploration of immune-related biological markers may assist in the clinical diagnosis, differential diagnosis, assessment of disease stratification, disease development, and prognosis of IPF will be a research hotspot.

Although the “disease related” and “innate immune mechanism” clusters were likely to receive less attention compared to the “biomarker related” and “inflammation related” clusters, these two clusters focused on “mechanisms”, “pathogenesis”, and “macrophages”, which were also closely related to immunity. This suggests that the study of IPF and immunity can provide a better understanding of the pathogenesis of IPF mechanisms.

To date, the etiology and mechanism of IPF remain clear, and the progression of IPF is variable, ranging from a chronic progressive stage for many years to a sudden acute exacerbation stage, which can be accompanied by various complications. So far, none of the medications for IPF have been able to reverse the process of fibrosis. In addition, there is no effective basis for early diagnosis, and it is necessary to integrate clinical symptoms, imaging, alveolar lavage fluid results, and even pathological biopsy to establish a diagnosis. By the time the diagnosis is confirmed, the disease is relatively serious, and the remaining survival time is very short. Furthermore, there is a lack of a standardized index to evaluate the effect of therapy. Based on these points, reliable and easily usable biological markers are urgently required to assist in early diagnosis, differential diagnosis, evaluation of disease severity, and evaluation of response to therapy, which could all be used as surrogate endpoints for clinical trials. Ideally, biological markers should be able to replace invasive methods, such as fiberoptic bronchoscopy and lung biopsy, as diagnostic tools. At present, the existing potential biomarkers for IPF are artificially classified into three categories according to their different biological characteristics as follows: those related to dysfunctional alveolar epithelial cells, those related to ECM remodeling and fibroblast differentiation, and those relevant to immune dysfunction [44, 45]. Although the hypothesis of inflammation in the pathogenesis of IPF has been questioned, it is generally accepted that inflammations, both innate and adaptive immune responses, are involved in the process and are essential to almost all wound healing and fibrosis processes, including IPF [13, 16]. Therefore, potential biomarkers of IPF related to immunity could also be involved in the innate and adaptive immune response systems.

Regarding the innate immune system, Prasse et al. [46] found that serum concentration of CC chemokine ligand-18 (CCL18) was associated with severity and mortality in IPF patients and it was used as the first biomarker to predict IPF mortality. CCL18, a marker of alveolar macrophage activation, mediated by Th2 cytokines [47, 48], promotes collagen production by fibroblasts [49] and has been used as a diagnostic biomarker for IPF [50]. Toll-like receptors (TLRs) are important protein molecules in innate immunity and are bridging linking innate immunity and adaptive immunity. The influence of L412F polymorphism on the innate immune recognition receptor TLR3 in IPF has attracted much attention. O'Dwyer et al. [51] found that TLR3 L412F was associated with lung function decline and high mortality in a bleomycin- (BLM-) induced pulmonary fibrosis mouse model. McElroy et al. [52] revealed that this effect might be because 412 F-heterozygous patients were likely to suffer from bacterial and viral infections. Using the single-cell gene sequencing dataset, Li et al. [53] confirmed that lung macrophages express Toll-interacting protein (TOLLIP) which is an inhibitory adaptor protein that acts downstream of TLRs [54]. Oldham et al. [55] discovered that TOLLIP polymorphisms affect the efficacy of N-acetylcysteine in patients with IPF, and further showed that different genotypes have opposite effects on patients. Moreover, Bonella et al. [56] confirmed that the minor allele of TOLLIP rs5743890 was associated with acute exacerbation and high mortality rate. Therefore, TOLLIP could be used as a biomarker to stratify disease severity, assess prognosis, and aid in the selection of treatment options. It should be noted that many other proteins, such as Defensins, S100A12, and Chitinase-3-like protein 1 (YKL-40), which are closely related to the innate immune system, were also found to be potential biomarkers for acute exacerbation, poor survival, and worse functional status in IPF [44, 45].

Studies have confirmed that many proteins related to the acquired immune system, such as anti-heat shock protein- (HSP-) 70 antibodies [57], C-X-C motif chemokine 13 (CXCL13) [58], anti-vimentin antibodies [59], and some T-cell subpopulations, might be associated with worse prognosis, acute exacerbations, and pulmonary hypertension. Thus, these proteins could be used as objective biomarkers in the diagnosis of IPF.

Ideal biomarkers for any pathology should be stable, accurate, sensitive and specific, and reproducible [50]. Although there were many biomarker candidates have been identified, none have met all of the conditions and addressed the needs of clinical and scientific research so far [45]. Therefore, in order to develop a more scientific and practical evaluation system, some researchers combined different types of biological markers, combined multiple potential biomarkers, or combined biomarkers with clinical data, achieving satisfactory benefits. For example, Clynick et al. [60] built a predictive model using osteopontin (OPN), polyamine spermidine (SPD), intercellular adhesion molecule-1 (ICAM1), and MMP7 to predict a patient's condition, and they applied OPN, MMP7, periostin (POSTN), and ICAM2 to predict the 1 year risk of exacerbation and death. Among these markers, ICAM1 and ICAM2 were associated with inflammation and immunity. These results were published in the *European Respiratory Journal* (IF = 33.795) in 2022. Due to the complexity of IPF, excavating or selecting ideal biomarkers to provide reliable clinical evidence and achieve individualized prediction is currently a research hotspot and will continue to be so in the future.

Of note, “regulatory T-cells” was a keyword that deserved attention in both frequency and time of co-occurrence in the IPF and immune literature, as shown using VOSviewer. Regulatory T cells (Tregs) play a crucial part in immune regulation and maintenance of immune tolerance. Similarly, researchers have found that Tregs may play exert different or even opposite regulatory effects through various signaling pathways at different stages of IPF. Reilkoff et al. [61] discovered that Tregs are increased in the peripheral blood and lung of IPF patients, which is positively correlated with disease progression. However, research by Kotsianidis suggested the opposite findings [62]. Although many current data are based on the analysis of the number of Tregs and their mechanism of action, and the conclusions obtained in mouse models and human samples are inconsistent, all findings suggest that Tregs do participate in the immune regulation of IPF. However, the mechanism remains unclear. Therefore, it is crucial to clarify the particular role of Tregs in IPF to study the pathogenesis and treatment of IPF.

### 4.3. Strengths and Limitations

In this study, publications related to IPF and immune response were retrieved from the WOS core database, which is a comprehensive core journal citation index database. This study objectively analyzed the information and characteristics of IPF and immune response publications in the past 22 years and predicted future research hotspots in this field. The authors aimed to provide a more intuitive reference for researchers in this field. Furthermore, all data were retrieved and downloaded within 1 day, avoiding the result bias caused by data updates. However, there were some unavoidable limitations of this study that should be acknowledged. First, in terms of the inclusion criteria, our search strategy only included publications in English. The types of literature selected in this study were only papers and review papers, while other forms, such as conference, abstract, and editorial materials, were ignored. Limitations to these inclusion criteria may have resulted in some missing data. Second, according to the current data extraction methods, the latest publications could not obtain high citation frequency and keyword co-occurrence frequency due to the time limitation, which could have affected our conclusions to some extent. Third, to avoid the influence of common interstitial pneumonia, publications with clear diagnostic criteria used since March 2000 were selected, which could have resulted in missing data before the time node that may have affected the analysis results. Finally, we only selected the WOS core database and ignored the literature in the WOS noncore database and other databases, which could also have impacted the results. Future work methods should incorporate more complete data, track the latest developments in the field, and conduct a more comprehensive analysis.

## 5. Conclusion

In summary, this study comprehensively evaluated the research trends and hotspots in IPF and immune response. The United States has contributed the most to this field, but China also had a considerable number of publications, showing a prominent growth trend. The *American Journal of Respiratory and Critical Care Medicine* could be referred to for the latest research and progress in this field. Kaminski N., Flaherty K. R., and Selman M. were the most academically influential authors in the field. The role of inflammation and immune response in IPF received more attention and thus exploration of ideal biological markers related to the immune response for diagnosis, disease stratification, curative effect evaluation, and prognosis assessment of IPF may be the research hotspot in the future. We hope that this study will encourage more researchers in medicine, molecular biology, and immunology to pay attention to the field of IPF and immune response to break clinical bottlenecks as soon as possible.

## Figures and Tables

**Table 1:** The top 10 most published authors in the field of IPF and immune response and their influences.

Author	Country	Institution	No. of publications	No. of citations	H-index
Kaminski N.	USA	Yale University School of Medicine	26	2,446	24
Bargagli E.	Italy	Siena University Hospital	17	539	10
Selman M.	Mexico	Instituto Nacional de Enfermedades Respiratorias Ismael Cosío Villegas	16	2,974	14
Flaherty K. R.	USA	University of Michigan	15	927	14
Martinez F. J.	USA	Weill Cornell Medical College	14	993	13
Pardo A.	Mexico	Universidad Nacional Autónoma de Mexico	14	2,898	12
Rottoli P.	Italy	University of Siena; University Hospital of Siena	13	619	11
Tzouvelekis A.	USA	Medical School, University of Patras	13	537	10
Gibson K. F.	USA	University of Pittsburgh School of Medicine	12	1,068	12
Noth I.	USA	University of Virginia	12	830	12

**Table 2:** The top 10 most cited publications in the field of IPF and immune response.

Title	Corresponding authors	Journal	IF	Publication year	Citations
Idiopathic pulmonary fibrosis: prevailing and evolving hypotheses about its pathogenesis and implications for therapy	Pardo A.	*Annals of Internal Medicine*	51.598	2001	1,304
Idiopathic pulmonary fibrosis	Hunninghake G. W.	*New England Journal of Medicine*	176.079	2001	725
Induction of epithelial–mesenchymal transition in alveolar epithelial cells by transforming growth factor-ss1: potential role in idiopathic pulmonary fibrosis	Borok Z.	*American Journal of Pathology*	5.77	2005	697
Mode of action of nintedanib in the treatment of idiopathic pulmonary fibrosis	Kolb M.	*European Respiratory Journal*	33.795	2015	410
Senolytics in idiopathic pulmonary fibrosis: results from a first-in-human, open-label, pilot study	Kirkland J. L.	*Ebiomedicine*	11.205	2019	412
Up-regulation and profibrotic role of osteopontin in human idiopathic pulmonary fibrosis	Kaminski N.	*Plos Medicine*	11.613	2005	326
Gene expression profiles distinguish idiopathic pulmonary fibrosis from hypersensitivity pneumonitis	Zlotnik A.	*American Journal of Respiratory and Critical Care Medicine*	30.528	2006	316
The role of bacteria in the pathogenesis and progression of idiopathic pulmonary fibrosis	Moffatt M. F.	*American Journal of Respiratory and Critical Care Medicine*	30.528	2014	317
TIMP-1, 2, 3, and 4 in idiopathic pulmonary fibrosis. A prevailing nondegradative lung microenvironment?	Pardo A.	*American Journal of Physiology Lung Cellular and Molecular Physiology*	6.011	2000	301
Fibroblasts from idiopathic pulmonary fibrosis and normal lungs differ in growth rate, apoptosis, and tissue inhibitor of metalloproteinases expression	Pardo A.	*American Journal of Respiratory Cell and Molecular Biology*	7.748	2001	273

## Data Availability

The datasets for this study are available from the corresponding author upon reasonable request.

## Abbreviations

AAY: Average appearing year
BLM: Bleomycin
CCL18: CC chemokine ligand 18
CFA: Cryptogenic fibrosing alveolitis
CXCL13: C-X-C motif chemokine 13
DCs: Dendritic cells
WOS: Web of science
ECM: Extracellular matrix
HSP: Anti-heat shock protein
ICAM1: Intercellular adhesion molecule-1
IF: Latest impact factor
ILC: Innate lymphocyte
IPF: Idiopathic pulmonary fibrosis
JCR: Journal citation reports
MMP: Matrix metalloproteinase
OPN: Osteopontin
PBMCs: Peripheral blood mononuclear cells
POSTN: Periostin
PSCHE: Pennsylvania commonwealth system of higher education
RRI: Relative research interest
SCI-E: Science citation index-expanded
SPD: Polyamine spermidine
TIMP: Tissue inhibitors of metalloproteinase
TLR: Toll-like receptor
TOLLIP: Toll-interacting protein
Tregs: Regulatory T cells
WOS: Web of science
WoSCC: Web of science core collection
YKL-40: Chitinase-3-like protein 1.

## Supplementary Materials

Supplementary 1 Details of the top 20 countries by coauthorship with at least 8 articles published.

Supplementary 2 Details of the top 18 institutions by coauthorship with at least 8 articles published.

Supplementary 3 Details of the top 19 journals by citations with at least 8 articles published.

Supplementary 4 Details of the 52 keywords that co-occurred at least 20 times.

Supplementary 5 Visual analysis of keywords in IPF and immune response using CiteSpace software.
