# Supplementary material for: Hotspots and Frontiers of Host Immune Response in Idiopathic Pulmonary Fibrosis: A Bibliometric and Scientific Visual Research from 2000 to 2022
Source: J Immunol Res. 2023 Apr 19;2023:4835710. doi: 10.1155/2023/4835710 (PMC10132898; doi:10.1155/2023/4835710)
Supplement: Supplementary 1 — Details of the top 20 countries by coauthorship with at least 8 articles published. [file 4835710.f1.doc]

**Supplemental table 1:** **Details of the top 20 countries by co-authorship with at least 8 articles published**

| **label** | **weight<Links>** | **weight<Total link strength>** | **weight<Documents>** | **weight<Citations>** | **score<AAY>** | **score<Avg. citations>** |
| --- | --- | --- | --- | --- | --- | --- |
| USA | 19 | 134 | 217 | 14,745 | 2014.1204 | 67.9493 |
| Peoples r China | 7 | 29 | 124 | 1,595 | 2018.75 | 12.8629 |
| Japan | 16 | 40 | 110 | 3,623 | 2012.6881 | 32.9364 |
| Italy | 16 | 52 | 62 | 1,742 | 2015.2787 | 28.0968 |
| England | 19 | 71 | 57 | 3,197 | 2013.0893 | 56.0877 |
| Germany | 15 | 67 | 47 | 2,004 | 2015.0435 | 42.6383 |
| Greece | 14 | 32 | 31 | 875 | 2011.6 | 28.2258 |
| South Korea | 5 | 15 | 25 | 826 | 2014.1667 | 33.04 |
| France | 15 | 32 | 20 | 558 | 2014.6 | 27.9 |
| Mexico | 15 | 27 | 17 | 2,975 | 2010.1176 | 175 |
| Netherlands | 16 | 23 | 16 | 422 | 2018.063 | 26.375 |
| Canada | 15 | 34 | 15 | 923 | 2014 | 61.5333 |
| Belgium | 15 | 37 | 14 | 354 | 2017.2308 | 25.2857 |
| Switzerland | 14 | 32 | 14 | 832 | 2015.0714 | 59.4286 |
| Finland | 5 | 9 | 12 | 403 | 2012.3333 | 33.5833 |
| Australia | 14 | 20 | 11 | 233 | 2017.0909 | 21.1818 |
| Brazil | 14 | 17 | 10 | 167 | 2013.1 | 16.7 |
| Scotland | 4 | 13 | 10 | 424 | 2014.2 | 42.4 |
| Spain | 16 | 30 | 10 | 459 | 2015.2222 | 45.9 |
| Sweden | 5 | 9 | 9 | 262 | 2015.2222 | 29.1111 |
