# Supplementary material for: Hotspots and Frontiers of Host Immune Response in Idiopathic Pulmonary Fibrosis: A Bibliometric and Scientific Visual Research from 2000 to 2022
Source: J Immunol Res. 2023 Apr 19;2023:4835710. doi: 10.1155/2023/4835710 (PMC10132898; doi:10.1155/2023/4835710)
Supplement: Supplementary 2 — Details of the top 18 institutions by coauthorship with at least 8 articles published. [file 4835710.f2.doc]

**Supplemental table 2:** **Details of the top 18 institutions by co-authorship with at least 8 articles published**

| **label** | **weight<Links>** | **weight<Total link strength>** | **weight<Documents>** | **weight<Citations>** | **score<AAY>** | **score<Avg. citations>** |
| --- | --- | --- | --- | --- | --- | --- |
| Univ Pittsburgh | 12 | 35 | 37 | 2,956 | 2014.324 | 79.8919 |
| Univ Michigan | 9 | 16 | 21 | 1,387 | 2011.619 | 66.0476 |
| Yale Univ | 5 | 14 | 18 | 1,174 | 2015.333 | 65.2222 |
| Univ Siena | 2 | 2 | 17 | 835 | 2013.941 | 49.1176 |
| Nagasaki Univ | 1 | 1 | 14 | 376 | 2011.857 | 26.8571 |
| Univ Nacl Autonoma Mexico | 6 | 9 | 14 | 2,898 | 2008.429 | 207 |
| Univ Calif san Francisco | 8 | 12 | 13 | 2,228 | 2013.923 | 171.3846 |
| Univ Colorado | 7 | 18 | 13 | 1,027 | 2013.308 | 79 |
| Mayo Clin | 2 | 3 | 12 | 1,069 | 2012.333 | 89.0833 |
| Univ Chicago | 7 | 17 | 12 | 808 | 2013.333 | 67.3333 |
| Univ Helsinki | 1 | 1 | 11 | 403 | 2011.455 | 36.6364 |
| Harvard Univ | 3 | 5 | 10 | 882 | 2011.7 | 88.2 |
| Natl Jewish Hlth | 9 | 20 | 10 | 537 | 2014.7 | 53.7 |
| Royal Brompton Hosp | 7 | 9 | 9 | 1,362 | 2014 | 151.3333 |
| Univ Edinburgh | 2 | 2 | 9 | 417 | 2013.444 | 46.3333 |
| Hannover Med Sch | 4 | 4 | 8 | 55 | 2020.75 | 6.875 |
| Nanjing Univ | 1 | 1 | 8 | 79 | 2018.75 | 9.875 |
| Vanderbilt Univ | 8 | 17 | 8 | 316 | 2015.25 | 39.5 |
