# Supplementary material for: Hotspots and Frontiers of Host Immune Response in Idiopathic Pulmonary Fibrosis: A Bibliometric and Scientific Visual Research from 2000 to 2022
Source: J Immunol Res. 2023 Apr 19;2023:4835710. doi: 10.1155/2023/4835710 (PMC10132898; doi:10.1155/2023/4835710)
Supplement: Supplementary 3 — Details of the top 19 journals by citations with at least 8 articles published. [file 4835710.f3.doc]

**Supplemental table 3: Details of the top 19 journals by citations with at least 8 articles published**

| **label** | **weight<Links>** | **weight<Total link strength>** | **weight<Documents>** | **weight<Citations>** | **score<AAY>** | **score<Avg. citations>** |
| --- | --- | --- | --- | --- | --- | --- |
| American Journal of Respiratory and Critical Care Medicine | 18 | 211 | 32 | 3723 | 2009.5312 | 116.3438 |
| American Journal of Physiology-Lung Cellular and Molecular Physiology | 15 | 55 | 14 | 1337 | 2014.4286 | 95.5 |
| European Respiratory Journal | 17 | 54 | 16 | 1289 | 2011.5 | 80.5625 |
| Plos One | 17 | 114 | 27 | 858 | 2015.6667 | 31.7778 |
| Respiratory Medicine | 18 | 88 | 26 | 848 | 2011.7692 | 32.6154 |
| American Journal of Respiratory Cell and Molecular Biology | 15 | 41 | 12 | 839 | 2010.6667 | 69.9167 |
| Chest | 16 | 44 | 14 | 732 | 2009.7857 | 52.2857 |
| Respiratory Research | 18 | 88 | 24 | 716 | 2015.5417 | 29.8333 |
| Thorax | 12 | 30 | 11 | 672 | 2011.9091 | 61.0909 |
| Journal of Immunology | 15 | 92 | 8 | 587 | 2009.875 | 73.375 |
| Respiration | 16 | 64 | 13 | 385 | 2008.7692 | 29.6154 |
| BMC Pulmonary Medicine | 12 | 31 | 13 | 262 | 2016.6923 | 20.1538 |
| Experimental Lung Research | 12 | 21 | 9 | 231 | 2014.8889 | 25.6667 |
| Sarcoidosis Vasculitis and Diffuse Lung Diseases | 12 | 31 | 12 | 212 | 2008.1667 | 17.6667 |
| Respirology | 11 | 21 | 8 | 187 | 2013 | 23.375 |
| Lung | 12 | 31 | 9 | 162 | 2014.2222 | 18 |
| Respiratory Investigation | 8 | 12 | 8 | 137 | 2018.75 | 17.125 |
| Frontiers in Immunology | 14 | 65 | 17 | 136 | 2020.1765 | 8 |
| Cells | 10 | 29 | 9 | 24 | 2021.4444 | 2.6667 |
