# Supplementary material for: Hotspots and Frontiers of Host Immune Response in Idiopathic Pulmonary Fibrosis: A Bibliometric and Scientific Visual Research from 2000 to 2022
Source: J Immunol Res. 2023 Apr 19;2023:4835710. doi: 10.1155/2023/4835710 (PMC10132898; doi:10.1155/2023/4835710)
Supplement: Supplementary 4 — Details of the 52 keywords that co-occurred at least 20 times. [file 4835710.f4.doc]

**Supplemental table 4:** **Details of the 52 keywords that co-occurred at least 20 times**

| **label** | **cluster** | **weight<Links>** | **weight<Total link strength>** | **weight<Occurrences>** | **score<AAY>** | **score<Avg. citations>** |
| --- | --- | --- | --- | --- | --- | --- |
| alveolar macrophages | 1 | 46 | 188 | 47 | 2011.5319 | 58.8936 |
| growth-factor-beta | 1 | 41 | 116 | 30 | 2011.6 | 111.5333 |
| cytokines | 1 | 35 | 103 | 31 | 2012.2333 | 31.4839 |
| epithelial-cells | 1 | 37 | 96 | 27 | 2013.4074 | 38.2222 |
| gene-expression | 1 | 46 | 146 | 44 | 2013.5227 | 75.9318 |
| cells | 1 | 48 | 242 | 71 | 2013.5286 | 44.5915 |
| lung | 1 | 45 | 199 | 54 | 2013.8269 | 34.2963 |
| protein | 1 | 28 | 53 | 23 | 2014.3182 | 26.6957 |
| mice | 1 | 34 | 68 | 21 | 2014.3333 | 30.1905 |
| pulmonary-fibrosis | 1 | 29 | 73 | 28 | 2014.4074 | 33.5714 |
| expression | 1 | 49 | 479 | 142 | 2014.4681 | 33.1056 |
| fibrosis | 1 | 36 | 85 | 27 | 2014.6667 | 43.8889 |
| receptor | 1 | 38 | 96 | 23 | 2014.913 | 25 |
| tgf-beta | 1 | 39 | 135 | 37 | 2015.4865 | 54.1351 |
| fibroblasts | 1 | 46 | 158 | 40 | 2015.55 | 29.6 |
| nf-kappa-b | 1 | 30 | 74 | 23 | 2015.6957 | 36.9565 |
| oxidative stress | 1 | 34 | 92 | 27 | 2015.963 | 32.5556 |
| bleomycin | 1 | 38 | 93 | 24 | 2016 | 35.25 |
| apoptosis | 1 | 42 | 114 | 31 | 2016.0968 | 40.6452 |
| lung fibrosis | 1 | 40 | 157 | 40 | 2016.2308 | 40.075 |
| activation | 1 | 50 | 210 | 57 | 2016.4464 | 39.6316 |
| inflammation | 1 | 50 | 276 | 76 | 2016.6533 | 24.8421 |
| usual interstitial pneumonia | 2 | 40 | 96 | 27 | 2011.741 | 96 |
| interstitial pneumonia | 2 | 46 | 181 | 55 | 2014.537 | 48.6727 |
| survival | 2 | 42 | 176 | 48 | 2015.1522 | 57.3125 |
| interstitial lung disease | 2 | 44 | 146 | 42 | 2015.3415 | 50.7381 |
| idiopathic pulmonary fibrosis | 2 | 51 | 816 | 252 | 2015.62 | 26.9722 |
| differentiation | 2 | 41 | 90 | 23 | 2016.2273 | 29.5652 |
| acute exacerbation | 2 | 48 | 188 | 52 | 2016.3922 | 28.1923 |
| pirfenidone | 2 | 42 | 141 | 36 | 2016.7714 | 39.8611 |
| interstitial lung-disease | 2 | 30 | 65 | 25 | 2016.84 | 43.96 |
| regulatory t-cells | 2 | 38 | 88 | 26 | 2016.9231 | 38.1538 |
| biomarkers | 2 | 34 | 107 | 23 | 2017.7391 | 22.5652 |
| prognosis | 2 | 32 | 89 | 22 | 2018.0455 | 22.6818 |
| diagnosis | 2 | 45 | 187 | 57 | 2018.2982 | 15.1228 |
| mortality | 2 | 34 | 98 | 26 | 2018.48 | 27.3077 |
| biomarker | 2 | 38 | 96 | 25 | 2018.64 | 23 |
| alveolitis | 3 | 36 | 82 | 23 | 2008.4783 | 44.5652 |
| sarcoidosis | 3 | 38 | 116 | 31 | 2010.2258 | 42.129 |
| bronchoalveolar lavage fluid | 3 | 44 | 158 | 40 | 2011.675 | 39.45 |
| bronchoalveolar lavage | 3 | 48 | 226 | 56 | 2011.7679 | 43.4464 |
| t-cells | 3 | 37 | 92 | 28 | 2013.7778 | 64.0714 |
| serum | 3 | 34 | 87 | 22 | 2013.9545 | 35.6364 |
| disease | 3 | 47 | 277 | 77 | 2014.1053 | 46.2857 |
| pathogenesis | 3 | 50 | 271 | 75 | 2015.9452 | 29.2667 |
| ipf | 3 | 47 | 206 | 58 | 2016.614 | 26.9828 |
| idiopathic pulmonary-fibrosis | 3 | 39 | 97 | 27 | 2016.7778 | 21.5185 |
| pulmonary fibrosis | 4 | 46 | 140 | 41 | 2012.7805 | 42.3659 |
| growth-factor | 4 | 37 | 127 | 32 | 2013.5938 | 47.5312 |
| macrophages | 4 | 47 | 177 | 46 | 2015.6522 | 29.1087 |
| cancer | 4 | 35 | 71 | 22 | 2016 | 30.9545 |
| mechanisms | 4 | 37 | 106 | 31 | 2016.2581 | 20.0968 |
