# Supplementary material for: Hotspots and Frontiers of Host Immune Response in Idiopathic Pulmonary Fibrosis: A Bibliometric and Scientific Visual Research from 2000 to 2022
Source: J Immunol Res. 2023 Apr 19;2023:4835710. doi: 10.1155/2023/4835710 (PMC10132898; doi:10.1155/2023/4835710)
Supplement: Supplementary 5 — Visual analysis of keywords in IPF and immune response using CiteSpace software. [file 4835710.f5.pdf]

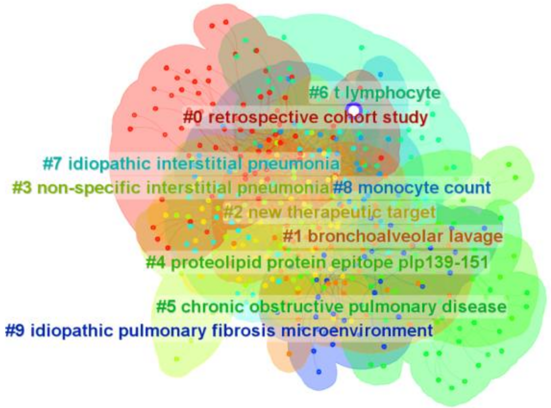

(a)

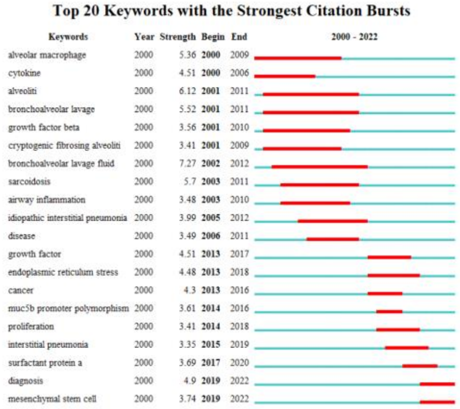

(b)

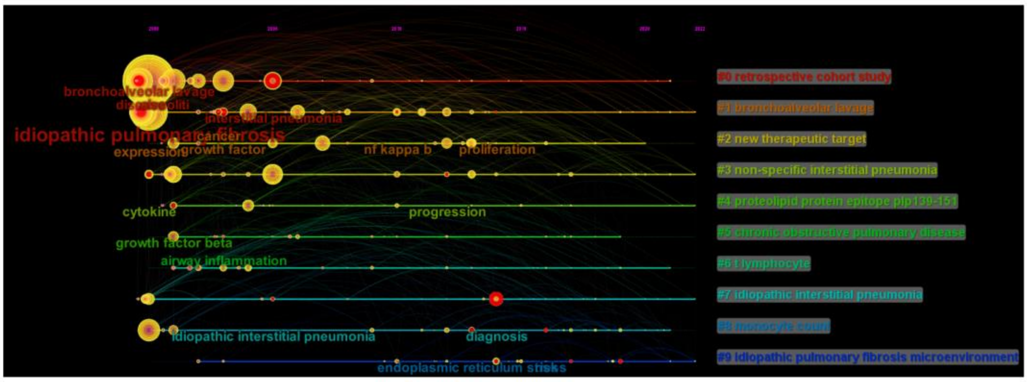

(c)

Supplemental Figures. Visual analysis of keywords in IPF and immune response using CiteSpace software. (A) Top 10 clusters with the largest number of keywords according to the LRR algorithm. Clusters are formed automatically according to the characteristics of keywords, and they are represented by different colors. The size of clusters is inversely proportional to the serial number. (B) Burst of the top 20 most frequently cited keywords. The blue line is the timeline, and the red part on the blue timeline represents the time zone of a particular keyword presentation. (C) A timeline map of keywords related to IPF and immune response. The position of the node on the horizontal axis represents the time when the keyword first occurs, and the size of the node represents the frequency of the keyword. The lines between the nodes represent co-occurrence. Color from blue to red indicates the time of occurrence from far to near.
